# Enhancing Salt Tolerance of Plants: From Metabolic Reprogramming to Exogenous Chemical Treatments and Molecular Approaches

**DOI:** 10.3390/cells9112492

**Published:** 2020-11-17

**Authors:** Manish Kumar Patel, Manoj Kumar, Weiqiang Li, Yin Luo, David J. Burritt, Noam Alkan, Lam-Son Phan Tran

**Affiliations:** 1 Department of Postharvest Science of Fresh Produce, Agricultural Research Organization, Volcani Center, Rishon LeZion 7505101, Israel; noamal@volcani.agri.gov.il; 2 Institute of Plant Sciences, Agricultural Research Organization, Volcani Center, Rishon LeZion 7505101, Israel; manojbiochem16@gmail.com; 3 Institute of Plant Stress Biology, State Key Laboratory of Cotton Biology, Department of Biology, Henan University, 85 Minglun Street, Kaifeng 475001, China; weiqiangli@henu.edu.cn; 4 Joint International Laboratory for Multi-Omics Research, Henan University, 85 Minglun Street, Kaifeng 475001, China; 5 School of Life Sciences, East China Normal University, Shanghai 200241, China; yluo@bio.ecnu.edu.cn; 6 Department of Botany, University of Otago, P.O. Box 56, Dunedin, New Zealand; david.burritt@otago.ac.nz; 7 Institute of Genomics for Crop Abiotic Stress Tolerance, Department of Plant and Soil Science, Texas Tech University, Lubbock, TX 79409, USA; 8 Stress Adaptation Research Unit, RIKEN Center for Sustainable Resource Science, 1-7-22, Suehiro-cho, Tsurumi, Yokohama 230-0045, Japan

**Keywords:** crop improvement, exogenous treatments, genetic engineering, primary metabolites, salt stress, secondary metabolites

## Abstract

Plants grow on soils that not only provide support for root anchorage but also act as a reservoir of water and nutrients important for plant growth and development. However, environmental factors, such as high salinity, hinder the uptake of nutrients and water from the soil and reduce the quality and productivity of plants. Under high salinity, plants attempt to maintain cellular homeostasis through the production of numerous stress-associated endogenous metabolites that can help mitigate the stress. Both primary and secondary metabolites can significantly contribute to survival and the maintenance of growth and development of plants on saline soils. Existing studies have suggested that seed/plant-priming with exogenous metabolites is a promising approach to increase crop tolerance to salt stress without manipulation of the genome. Recent advancements have also been made in genetic engineering of various metabolic genes involved in regulation of plant responses and protection of the cells during salinity, which have therefore resulted in many more basic and applied studies in both model and crop plants. In this review, we discuss the recent findings of metabolic reprogramming, exogenous treatments with metabolites and genetic engineering of metabolic genes for the improvement of plant salt tolerance.

## 1. Introduction

Environmental stresses (ESs), both abiotic and biotic, negatively impact plant growth and development and reduce crop yields by perturbing metabolic homeostasis [1,2,3]. Soil salinity is a major abiotic factor limiting crop yields worldwide, and is becoming a greater threat to global food security due to uneven rainfall, inundation of coastal lands with seawater, poor-quality water for irrigation due to groundwater depletion and degradation of high-salt rocks [3,4]. In general, the range of salt concentrations in water for irrigation purposes is between 0.6 to 1.7 dS/m [5,6]. Globally, it is estimated that about 6% of all land areas are affected by salt, with approximately 22% of cultivated and 33% of irrigated fields used for agriculture [7,8].

High soil salinity can cause salt stress (SS), which impairs many critical cellular functions in plants by disturbing various physiological, biochemical and metabolic processes [9]. However, many plant species have evolved strategies to tolerate SS, and thus, can grow in saline soils [10]. Upon exposure to excess salt, plants first sense the potential stressor and then activate a signaling network and a multifaceted response, which includes the synthesis of a range of compounds that help reduce the impacts of high soil salinity and maintain cellular homeostasis [11,12]. The plant cell wall is a complex structure that executes many functions throughout the life cycle of a plant. The cell wall of plants is critically important to the maintenance of cell shape by resisting internal hydrostatic pressures and protecting the cells in responses to ESs [13]. Primary metabolites (PMs) are involved in plant growth and development, whereas secondary metabolites (SMs) are derivative of PMs, and both PMs and SMs play key roles in plant adaptation to ESs, including SS [1,14]. However, concerning the metabolic changes that occur in plants responding to ESs, a lack of detailed information limits our understanding of how plants respond to ESs, especially SS.

Several approaches, namely chemical priming and genetic engineering, have been employed to enhance plant SS tolerance [15,16]. Plants can be more tolerant to SS through chemical priming. Priming agents such as natural metabolites/compounds or synthetic compounds have shown an excellent opportunity to increase salt tolerance in various models and important agronomic crop plants without modification of their genome. Moreover, current efforts have also been made in plant genetic engineering strategies to enhance plant tolerance to different types of abiotic stresses (ASs), including SS, based on the alteration of expression levels of genes that are associated with osmoregulation, metabolic pathways and metabolites [16]. The genetic engineering approach provides opportunities to increase the SS tolerance in crops by the activation of various signaling pathways participating in stress perception, signal transduction, osmotic regulation and production of antioxidant enzymes [17,18]. Agronomical important crops treated with priming agents can activate several physiological and biochemical processes, thereby enhancing SS tolerance, suggesting the potential applications of priming agents in crop stress management [15]. Moreover, identifying the functions of a particular gene or set of genes and associated endogenous metabolites will further help explore the mechanisms controlling complex physiological, biochemical and phenotypic traits [16,19]. In addition, manipulation of metabolic genes and application of priming agents can increase plant stress tolerance by changing the levels of associated transcripts, metabolite production and enzyme levels for membrane lipid biosynthesis [16,20]. In this review, we highlight recent research progress to uncover the metabolic reprogramming in plant response to SS, as well as the potential of priming treatments of seeds and plants with exogenous metabolites and the promising uses of genetic engineering in improving SS tolerance in commercially important crops. 

## 2. Metabolic Reprogramming in Plants Responding to SS

**Figure 1:**
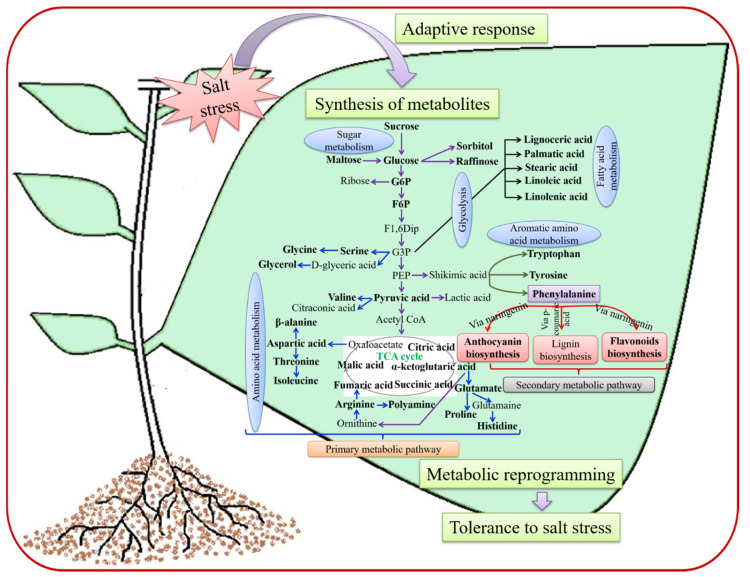
A simplified model of metabolic reprogramming in plants under salt stress. When plants sense high salinity, they undergo a metabolic reprogramming that involves changes of primary and secondary metabolites for maintaining appropriate osmotic homeostasis and activation of signaling pathways. This is the simplest strategy that plants use to acclimate to survive under salt stress. The bold metabolites are discussed in the text and they have important roles during salt tolerance. Purple arrows, sugar metabolism; blue arrows, amino acid metabolism; olive green arrows, aromatic amino acid metabolism; black arrows, fatty acid metabolism; red arrows, secondary metabolic pathways. F6P, fructose 6-phosphate; F1,6Dip, fructose 1,6-diphosphate; G6P, glucose 6-phosphate; G3P, glyceraldehyde 3-phosphate; PEP, phosphoenolpyruvate; TCA, tricarboxylic acid.

Various ESs, such as salinity, drought and high temperatures, can lead to the hyper-accumulation of a wide range of metabolites in plants [21]. The tolerance ability of plants against SS is typically based on their capacity to maintain a proper level of primary and secondary metabolic processes and defense responses [22] (Figure 1). During the progression of SS, plants produce PMs and SMs as excretory products, which secrete from shoots, roots and leaves at different stages of plant development [22] (Table 1). As metabolites are the end products of various cellular processes, the plant metabolome is often considered to be the bridge between a plant’s genotype and phenotype [23]. Thus, metabolomic analysis can link the genotypic and phenotypic changes that occur in plants responding to SS, and help to investigate and identify key differences between SS-tolerant and SS-sensitive plant species/genotypes [24,25]. To understand the metabolic reprogramming under SS, two general approaches have been used: targeted and non-targeted metabolomics. Targeted metabolomics is a tool for the identification, estimation, and interpretation of specific or known metabolites in plants under stress [26,27]. On the other hand, non-targeted metabolomics can produce a global overview of the most abundant metabolites found in plants under SS, when compared to unstressed control plants [9,28]. Therefore, the use of plant metabolomics to study the changes in both PM and SM levels is important for our understanding of metabolic reprogramming in plants during stress, and the chemical and biotechnological applications of this knowledge for SS management of crop plants [29,30,31].

### 2.1. Primary Metabolites and Their Response to SS

PMs are essential for the normal functioning of plant cells and are directly involved in various biochemical and physiological processes, e.g., photosynthesis and respiration, providing the energy and the precursors required for the biosynthesis of new macromolecules necessary for developmental processes in plants [32,33]. PMs include sugars (mono-, di- and trisaccharides), polyols (e.g., sorbitol and mannitol) and amino acids (AAs) such as proline, which can serve as osmolytes and osmoprotectants in plants under ASs [11,34].

#### 2.1.1. Carbohydrates: Compatible Solute Accumulation under SS

SS adversely affects carbohydrate metabolism in plants, and the accumulation of sugars and polyols plays a vital role in osmotic adjustment, carbon storage and free radical scavenging [35]. Plants under SS accumulate different soluble sugars, e.g., sucrose, trehalose and raffinose, and sugar alcohols, e.g., sorbitol and mannitol, to control osmotic stress levels, maintain cell turgor pressure and help stabilize cell membranes [35,36] (Figure 1). The synthesis of compatible solutes/osmolytes is the simplest metabolic stress acclimation response observed in plants. Carbohydrates, such as hexoses (fructose and glucose), disaccharides (sucrose and trehalose) and oligosaccharides (raffinose and stachyose), are important osmolytes reported in various studies [35,37] (Figure 1). In *Arabidopsis thaliana*, trehalose regulates the ionic balance and cellular redox state under high salt concentrations [38]. Similarly, enhanced intracellular levels of raffinose and galactinol in *Arabidopsis* were reported by Nishizawa et al. [39] in response to methyl viologen treatment, salinity or chilling stress. Additionally, the authors observed that both galactinol and raffinose played important roles in protecting plants against oxidative damage by scavenging hydroxyl radicals [39,40].

Soluble sugars can also regulate plant metabolism by modulating the expression of sugar-sensitive genes and the activities of some enzymes under ESs [41]. Sucrose and hexose sugars can act as signaling molecules and play a dual function in the regulation of gene expression by down-regulating stress-related genes and up-regulating growth-related genes [41]. Both growth- and stress-related genes are regulated through hexokinase (HXK)-dependent and/or HXK-independent pathways [41]. In addition, plants also possess sucrose-non-fermenting-1 (SNF1)-related protein kinases (SnRKs) that are implicated in sugar sensing and also have a role in the interface between metabolic and stress signaling pathways [42,43]. Some key examples demonstrating the importance of PMs in plants exposed to SS are detailed below. 

Under SS, wild and cultivated barley showed different metabolic responses in roots and leaves. Tibetan wild barley (*Hordeum spontaneum*) had a higher compatible solute concentration compared with cultivated barley (*H*. *vulgare*), and cultivated barley showed increased glycolysis and energy consumption under high salinity as compared with wild barley [44]. Two chickpea (*Cicer arietinum*) varieties (Genesis 836 and Rupali) subjected to a salinity treatment displayed increased levels of sugar alcohols, including erythritol, xylitol, arabitol, mannitol, galactitol and inositol, clearly indicating the important roles of these molecules in salt tolerance [45]. In addition, sucrose and inositol were shown to be accumulated in the leaves of *Atriplex halimus* plants under high salinity [46] (Table 1). In *Casuarina glauca* subjected to SS, glucose, sucrose, fructose and trehalose remained unchanged in the nodules at lower level of NaCl (200 mM), whereas trehalose significantly increased in the roots at both low (200 mM) and high levels (400 and 600 mM) of NaCl [47]. The authors also observed that levels of the AAs serine, glycine, valine, alanine, proline, glutamine, arginine and glutamate remained unaltered in the nodules, whereas levels of these AAs decreased in the roots at 200 mM NaCl [47]. These results indicated that the primary metabolome of roots and nodules had diverse metabolite responses to both low and high salt concentrations, with the more noteworthy changes being found in roots [47]. In sugar beet (*Beta vulgaris*), arabinose, mannitol, gluconolactone, inositol, serine, proline and thymine showed significant accumulation after both 3 h and 14 days of SS. On the other hand, galactose, putrescine, trehalose, sucrose, homocysteine, norleucine, cytosine, xylose and glycolate contents first increased in response to SS, but then decreased at the later time point of SS [48]. In response to SS, the contents of five sugars, gentiobiose, fructose, fucose, mannose and trehalose, increased in Vlamingh barley variety, whereas the levels of gentiobiose, fructose and glucose increased in Sahara variety [49]. Thus, alterations in the levels of metabolites associated with energy storage under SS suggest that regulation of carbohydrate metabolism is crucial for SS tolerance in many plants, including crop plants.

#### 2.1.2. Amino Acid Production and Their Involvement in Plants under SS

AAs are important metabolites in plants not only for protein synthesis and other key cellular functions [35], but they also act as important osmolytes to balance the cellular osmotic potential and control ion transport, as well as function as scavengers of reactive oxygen species (ROS) generated in plants under SS [50] (Figure 1). For example, proline is widely accepted as an osmolyte that accumulates and protects plant cells from salinity-induced damage (Figure 1). The importance of changes in AA levels, and the coordination of AA metabolism in plants under SS were demonstrated using four barley genotypes (CM72, Gairdner, XZ16 and XZ169) [44]. In response to SS, proline levels increased in all four genotypes, but changes in the levels of other AAs, e.g., alanine, aspartate, glutamate, threonine and valine, were genotype-dependent [44]. In a similar study of two genotypes *Glycine max* (C08) and *G. soja* (W05) grown under SS, it was noted that the alanine content decreased in the seedling leaves of both genotypes, while serine and glycine levels increased in the W05 genotype only [51]. Furthermore, Cao et al. [49] reported that the concentrations of eight AAs and amines increased significantly in all barley varieties under SS, including 4-hydroxy-proline, asparagine, alanine, arginine, phenylalanine, citrulline, glutamine and proline. Some other AAs exhibited changes only in specific varieties [49]. In addition, AA profiling of cumin (*Cuminum cyminum*) plants showed that the levels of most AAs (except asparagine) increased in plants under SS, compared with that in control plants [9]. Metabolomics studies have also been conducted on the halophytic species *Aeluropus lagopoides* and *Salicornia brachiata* under SS [28,52]. Under SS, *A. lagopoides* showed changes in the levels of metabolites. The levels of some AAs, e.g., proline, alanine, valine, asparagine, arginine, lysine, histidine, glutamine, phenylalanine, glycine, tyrosine, serine and cytosine, increased, whereas the levels of some TCA cycle-related metabolites, e.g., aconitate, citrate, succinate, 2-oxoglutarate and fumarate, remarkably decreased [52]. A metabolomic study of the succulent halophyte *S. brachiata* plants under SS showed an increase in the contents of some AAs, e.g., asparagine, valine, cysteine, proline, lysine, leucine, isoleucine, methionine and tyrosine, and also of some fatty acids, e.g., oleic acid, stearic acid, α-linolenic acid, linoleic acid and lignoceric acid [28]. Furthermore, under SS, 19 metabolites were identified in extracts of *S. corniculata* leaves [53]. Out of nine AAs (valine, glycine, alanine, leucine, isoleucine, glutamine, glutamate, aspartate and threonine) and three sugars (sucrose, glucose and fructose) [53], only sucrose and alanine displayed significantly different levels under SS, while other metabolites showed no significant differences in their contents under SS [53]. Under SS conditions, the levels of AAs varied significantly among three salinity-tolerant lines (G58, G1710 and IR64) and two salinity-susceptible lines (G45 and G52) of rice [54]. Proline, isoleucine, phenylalanine and leucine contents increased among the identified AAs in all five investigated lines. Moreover, the contents of proline, valine and tyrosine were lower in three salinity-tolerant lines than in two salinity-susceptible lines. Compared with the control, most of the AAs increased in response to SS in all five lines [54]. Furthermore, a recent study showed that proline, valine, cysteine, aspartic acid, glutamine, ornithine and citrulline levels increased, while serine, threonine, alanine, glutamate, leucine, glycine and α-aminoadipic acid levels decreased in cucumber seedlings exposed to SS [55]. 

### 2.2. Secondary Metabolites and Response to SS

SMs are generally not required for the normal functioning of plant cells, but have important functions in protecting plants against biotic stresses and ASs (Figure 1). Plant species show considerable variation in the types and levels of SMs that are produced in responses to stresses [71,72,73]. It has been estimated that there are more than 100,000 SMs within the plant kingdom, and these are classified into three main groups, nitrogen-containing compounds (e.g., alkaloids and glucosinolates), terpenes and phenolic compounds (e.g., phenylpropanoids and flavonoids) [31,74,75] (Table 1; Figure 1). The levels of SMs found in plants may alter in response to SS-induced osmotic stress and/or ion toxicity [71]. Some examples demonstrating the importance of SMs in plant response to SS are detailed below.

#### 2.2.1. Alkaloids: As Stimulants under SS

The naturally occurring alkaloids, which contain a nitrogen atom in a heterocyclic ring, are SMs that have antioxidant activities and play important roles as ROS scavengers under SS [76,77]. Sachan et al. [78] showed that ROS levels are also involved in regulating alkaloid pathway in undifferentiated *N. tabacum* cells. The content of alkaloid reserpine in *Rauvolfia tetraphylla* increased when cells were exposed to SS [79]. Jaleel et al. [80] reported that the application of 80 mM NaCl increased the contents of indole alkaloids as compared with control plants (unstressed) in *C*. *roseus*, while Osman et al. [81] found that in the shoots of *C*. *roseus* plants exposed to 150 mM NaCl for 2 months, the content of vincristine significantly accumulated as compared with the control [81]. Furthermore, Benjamin et al. [82] observed the levels of numerous alkaloids, such as 3,6-dihydronicotine, portulacaxanthin II, papaveroxine and secoberbine, increased, while the contents of alkaloids, such as harmol and ricinine, decreased in *S*. *brachiata* leaves during SS. In leaves of *Sesuvium portulacastrum* treated with 200 mM NaCl, the contents of some alkaloids, such as cyclo-dopa 5-*O*-glucoside, *N*-formyldemecolcine and colchicine, increased, whereas those of castanospermine, cyclo-acetoacetyl-l-tryptophan and chelirubine decreased [82]. On the other hand, the levels of many alkaloids, such as chelirubine, deoxypumiloside, 2-descarboxy-betanidin and noscapine, were reduced in roots of *S*. *maritima* under SS [82].

#### 2.2.2. Terpene Production and Response to SS

Plant terpenoids (e.g., isoprene-C5, monoterpenes-C10, sesquiterpenes-C15, diterpenes-C20 and polyterpenoids-C5xn) are naturally occurring chemicals and have diverse functions in plant growth and development [83]. Terpenoids play important ecological roles in the interactions between plants and ESs [84]. Different types of stresses can promote or inhibit the production of terpenes [85]. Additionally, it is well established that many stressors induce a simultaneous increase of ROS, leading to oxidative stress and triggering signaling pathways toward metabolic reprogramming [71]. Some studies showed that terpenes exhibit antioxidant activities [86,87], suggesting their functions in overcoming oxidative stress [85]. Karray-Bouraoui et al. [88] observed that SS enhanced the contents of pulegone, while that of neomenthol was not affected in shoots of *Mentha pulegium*. Recently, Valifard et al. [69] examined the leaves of *Salvia mirzayanii* plants exposed to SS and found that the concentrations of the terpenoids, such as 1,8-cineole and linalyl acetate, increased, while that of bicyclogermacrene decreased. The authors also isolated the *cineole synthase1* gene (*SmCin1*), which plays a key role in the biosynthesis of the essential oil 1,8-cineole, and showed that its expression was induced in leaves by SS [69]. In roots of *S*. *brachiata* treated with SS, increased levels of oleanolate 3-β-d-glucuronoside-28-glucoside, taxol and glycyrrhetinate terpenoids were observed, while in the stressed leaves accumulations of sesquiterpenoids such as desoxyhemigossypol-6-methyl ether, costunolide, heliespirone C and 15-hydroxysolavetivone were noted [82]. On the other hand, in roots of *S*. *portulacastrum* exposed to SS, triterpenoids such as amyrins and betulinic acid decreased [82].

#### 2.2.3. Phenolics: Potential Antioxidants under SS

The phenolics, e.g., phenylpropanoids, flavonoids, tannins, coumarins and lignins, form an important class of SMs in plants [89]. Flavonoids are a large group of naturally occurring phenolics, including flavones, isoflavones, flavonols, flavanones, proanthocyanidins, anthocyanidins and chalcones [90,91]. They are important phytochemicals and have many novel roles in plants [92]. Kováčik et al. [63] observed significant accumulation of various phenolics, including protocatechuic acid in leaf rosettes, and chlorogenic and caffeic acids in the roots of *Matricaria chamomilla* in responses to SS. Lim et al. [64] found that various concentrations of NaCl induced the accumulation of four different phenolic compounds, i.e., rutin, orientin, isoorientin and vitexin, in the sprouts of buckwheat (*Fagopyrum esculentum*). These results support the view that NaCl treatment can enhance the nutritional contents of sprouts, including the levels of phenolic compounds. Furthermore, the contents of phenolic compounds such as oleuropein were found to be increased, while that of hydroxytyrosol decreased in the leaves of four olive (*Olea europaea*) cultivars grown under SS (125 mM) [67]. Additionally, SS can modulate the contents of various phenolic compounds in both tolerant and susceptible rice varieties [93]. The contents of protocatechuic acid and vanillin increased in tolerant varieties, whereas they decreased in a susceptible cultivar. Moreover, *p*-coumaric and ferulic acids were detected only in tolerant rice varieties, suggesting that they have a role in rice tolerance to SS [93]. Differentially produced phenolics, including luteolin, salvianolic acid, kaempferol and quercetin, were also identified in cumin at various salt concentrations [9]. The biosynthesis of flavonoids is up-regulated in response to a wide range of abiotic and biotic stresses, including defense responses to pathogens, cold, drought and SS [94,95,96]. Thus, increased flavonoid levels may help plants counter stress-induced oxidative damage [94,97]. Some flavonoids can act as antioxidants and scavenge ROS generated in plants under oxidative stress because of the presence of structures such as the dihydroxy B-ring-substituted flavonoid glycosides [97]. The contents of two flavonols, i.e., quercetin and kaempferol, were significantly higher in *Apocynum venetum* seedlings under SS [98]. Moreover, the flavonol biosynthesis-related genes, i.e., *flavonoid 3′-hydroxylase* (*AvF3′H*), *flavanone 3-hydroxylase* (*AvF3H*) and *flavonol synthase* (*AvFLS*), were up-regulated, while *chalcone synthase* (*AvCHS*), *chalcone-flavonone isomerase* (*AvCHI*) and *flavonol 3-O-galactosyltransferase* (*AvF3GT*) were down-regulated under SS [98]. Oliveira et al. [99] reported that in response to SS, maize (*Zea mays*) cell walls showed reduced deposition of matrix polysaccharides, cellulose and lignin in seedling roots, as well as roots and stems of plants. The authors also observed that the contents of arabinoxylans reduced in the roots of salt-stressed seedlings and plants [99]. Moreover, the authors provided new insights into salt-induced modulation in the activities of enzymes, such as *p*-hydroxycinnamate-CoA ligase, hydroxycinnamaldehyde dehydrogenase and feruloyl esterase, and expression of their coding genes required for ferulic acid biosynthesis and cell wall feruloylation [99]. In addition, Pi et al. [100] reported that dihydroxy B-ring-substituted flavonoids, such as cyanidin 3-arabinoside chloride, luteolin 3′-methyl ether 7-glucuronosyl-(1→2)-glucuronide, quercetin 3-(6″-methylglucuronide), cyanidin 3-(6″-succinyl-glucoside) and quercetin 3,3′,7 -tri-O-sulfate, significantly increased in soybean roots under SS.

#### 2.2.4. Dimethylsulfonium Compounds: An Important Osmoprotectant

Dimethylsulfonium compounds such as GB can play an important role under SS, with GB being one of the most common metabolites found in plants exposed to SS [101]. GB plays important roles in osmoprotection and ROS scavenging, and also contributes to the maintenance of protein integrity by inhibiting protein carbonylation [102,103]. When GB is present at high levels, together with proline, it is so effective in protecting plants against ROS-induced oxidative stress under SS [104,105]. In addition, GB improves the activities of glutathione-*S*-transferase and glutathione peroxidase in plants, which can help minimize the lipid peroxidation of cellular membranes caused by oxidative stress [102,106]. Most importantly, GB accumulates in many crop plants, e.g., barley and rice, and in halophytes, such as *Suaeda maritima* and *Avicennia marina*, under SS [56,70,106,107,108]. Gavaghan et al. [57] reported that the contents of GB, sucrose and asparagine in maize shoots increased, while the γ-aminobutyric acid, malic acid, aspartic acid and *trans*-aconitic acid contents in roots increased in response to high salinity. A partial least squares-discriminant analysis (PLS-DA) of ^1^H NMR spectral data revealed that the progressive metabolic response was higher in shoots compared to roots [57]. GB precisely rises its own content and improves the action of antioxidant enzymes, such as ascorbate peroxidase (APX), catalase (CAT) and superoxide dismutase (SOD), while reducing the contents of malondialdehyde (MDA) and H_2_O_2_ in *Lolium perenne* under SS [109]. In another study conducted in *N. tabacum* plants, short-term and low-dose SS induced metabolic shifts toward gluconeogenesis, which involved accumulation of mainly glucose, sucrose and fructose with depletion of pyrimidine and purine metabolites, while a high-dose of salt increased the accumulation of the osmolytes proline, myo-inositol and GABA [61]. Dimethylsulfoniopropionate (DMSP) is produced by a large diversity of macroalgae [110] and has a significant impact on SS and temperature shifts [111,112]. Intracellular functions of DMSP are not well understood but this molecule has been proposed to act as an osmoregulator [113] and antioxidant [114]. Wittek et al. [112] conducted a controlled experiment with *Fragilariopsis cylindrus* and found an increase in the concentrations of particular DMSP and dimethylsulfoxide (DMSO) with increasing salt concentrations up to 100 mM. These results suggested that DMSP and DMSO might play a role in SS responses and help *F*. *cylindrus* to counterattack increased salinity levels [112]. The above studies and the additional studies detailed in Table 1 demonstrate that reprogramming of PMs in plants are an adaptive response for survival under SS.

## 3. Improvement of SS Tolerance by Exogenous Treatments with Metabolites

**Figure 2:**
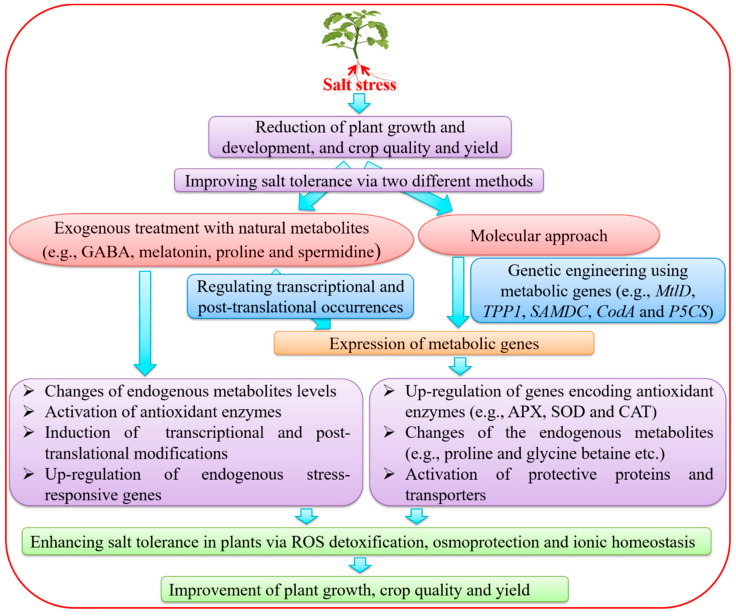
Approaches to improve salt tolerance via treatments with exogenous natural metabolites and genetic manipulation in plants. Treatments of plants using exogenous metabolites enhance salt tolerance by activation of antioxidant enzymes, changes of endogenous metabolites, up-regulation of stress-responsive genes and induction of transcriptional and post-translational modifications. Modulation of expression of metabolic genes in plants may lead to the improvement of salt tolerance by the up-regulation of genes encoding antioxidant enzymes and activation of transporters and protective proteins. APX, ascorbate peroxidase; CAT, catalase; *CodA*, *choline oxidase*; GABA, γ-aminobutyric acid; *MtlD*, *mannitol-1-phosphate dehydrogenase*; *P5CS*, *∆^1^-pyrroline-5-carboxylate synthetase*; ROS, reactive oxygen species; *SAMDC*, *s-adenosylmethionine decarboxylase*; SOD, superoxide dismutase; *TPP1*, *trehalose-6-phosphate synthase1*.

Over the last decades, many studies of exogenous treatments have been reported for improvement of plant tolerance to different types of ASs, including SS, using natural and synthetic metabolites/compounds [115]. Exogenous treatment is a method in which seeds or plants are treated with different metabolites to protect them against SS (Figure 2, left branch). Different types of naturally occurring metabolites, such as non-proteinogenic AAs, AAs, antioxidant enzymes, hormones, sugar, vitamins and polyamines (PA), have been used in various exogenous treatments of plants (Table 2). The exogenous treatments with these metabolites can protect plants from SS by osmoregulation, and ROS and methylglyoxal detoxification [15]. Due to their defensive effects, treatments with metabolites may play an important role in the regulation of transcriptional and post-translational occurrences [15]. Melatonin is a signaling molecule involved in different physiological roles in plant growth, development and responses to ESs [116]. Exogenous treatment of melatonin mitigated the tolerance of wheat (*Triticum aestivum*) seedlings against SS as judged by the increases in dry shoot weight, chlorophyll content, leaf photosynthesis rate and indole-3-acetic acid content, and reduction of H_2_O_2_ levels [117]. Furthermore, exogenous melatonin treatment increased endogenous melatonin content in *T*. *aestivum* seedlings by inducing expression levels of *TaSNAT* gene that codes for a regulatory enzyme involved in the biosynthesis of melatonin [117]. In a recent study of tomato (*Solanum lycopersicon*) seedlings, melatonin treatment increased plant growth, chlorophyll *a* and *b* contents, total soluble carbohydrate content and increased the activities of carbonic anhydrase and ribulose-1,5-bisphosphate carboxylase/oxygenase under SS [118]. Additionally, melatonin treatment improves osmoregulation by increasing total soluble carbohydrate content, proline and Δ^1^-pyrroline-5-carboxylate synthetase (P5CS) activity [118]. 

5-aminolevulinic acid (ALA) is a precursor of tetrapyrroles and plays a vital role in plant stress adaptation. ALA improved the antioxidant enzyme activities and photosynthesis, and increased the accumulation of chlorophylls and hemes in *Brassica napus* seedlings under SS [126]. Exogenous ALA induced the concentration of proline and up-regulated the expression of *P5CS* and *proline dehydrogenase* (*ProDH*) genes encoding proline metabolic enzymes in *B. napus* seedlings treated with NaCl [126]. Bajwa et al. [123] found that seed priming with sorghum extracts and benzyl aminopurine increased SS tolerance of *T*. *aestivum*. Treatment of salt-sensitive muskmelon (*Cucumis melon*) with exogenous γ-aminobutyrate mitigated the effects of saline-alkaline stress by enhancing chlorophyll biosynthesis, and more importantly by inducing the production of H_2_O_2_ that might act as a signaling molecule [127]. Exogenous treatment with proline (30 mM) mitigated the adverse effects of SS in sorghum plants by altering proline metabolism and expression of *P5CS1* and *ProDH* [139].

PAs are aliphatic nitrogenous compounds with low molecular weight in plants, which are crucial for growth and development and play important roles in plant responses to ESs due to their polycationic nature [140]. In higher plants, PAs are mainly present in three forms, putrescine (Put), spermidine (Spd) and spermine (Spm) [141]. Under SS, exogenous application of Put to cucumber (*Cucumis sativus*) plants was reported to regulate carbohydrate metabolism, and the levels and ratios of endogenous hormones, thereby improving the photosynthesis and growth [131]. Two zoysiagrass (*Zoysia japonica*) cultivars that were SS-sensitive (cv. Z081) and SS-tolerant (cv. Z057) were treated with different concentrations of exogenous Spd, and subjected to 200 mM SS [125]. Both cultivars shared similar types of responses in various physiological and biochemical assays and PA metabolism under SS with increasing exogenous Spd concentration. In response to SS, ornithine decarboxylase, diamine oxidase and S-adenosylmethionine decarboxylase activities increased in both cultivars with exogenous Spd treatment [125]. In addition, exogenous Spd treatment reduced MDA and H_2_O_2_ levels, induced antioxidant enzyme activities, and improved the tolerance of zoysiagrass plants to SS [125]. Taken together, exogenous treatments of seeds/plants with metabolites is a potential approach for plant stress management for improvement of plant growth and productivity under various ESs (Figure 2, left branch).

## 4. Genetic Engineering of Metabolic Genes for the Improvement of Salt Tolerance

Plant genetic engineering approaches for SS tolerance are based on modulation of enzymes that are involved in the synthesis of functional metabolites [142], antioxidant enzymes, enzymes for membrane lipid biosynthesis and transporters [143] (Figure 2, right branch; Table 3). *ProDH* and *P5CS* genes play important roles in SS tolerance by regulating the proline synthesis [16]. Karthikeyan et al. [144] reported that ectopic expression of *P5CS* gene of *Vigna aconitifolia* increased tolerance of transgenic rice plants to SS. The overexpression of *P5CS* gene from *A. thaliana* increases proline accumulation and improved salt tolerance in transgenic potato (*Solanum tuberosum*) plants [145]. Ectopic expression of *P5CSF129A* gene of *V. aconitifolia* exhibited higher proline accumulation and better root growth in transgenic rice plants under SS [146]. Introduction of the *arginine decarboxylase* (*ADC*) gene from *Avena sativa* into *Lotus tenuis* conferred SS tolerance by producing more proline, which stabilized the cell membrane [147]. Nevertheless, ectopic expression of *ADC* gene from *Datura stramonium* in rice enhanced tolerance of transgenic plants to drought by increasing the levels of putrescine during the stress [148], suggesting the potential application of the *ADC* gene in conferring tolerance to multiple stresses [148]. Transgenic wheat plants harboring the *choline dehydrogenase (betA)* gene of *Escherichia coli* were more tolerant to SS due to an increase of GB content in transgenic plants [149]. Transforming cotton (*Gossypium hirsutum*) with the *choline monooxygenase (AhCMO)* gene of *Atriplex hortensis* increased the content of GB in transgenic plants, providing a better protection of the cell membrane under SS [150] (Table 3).

Introduction of the *cysteine protease* (*SmCP*) gene from *Salix matsudana* into *A*. *thaliana* enhanced SS tolerance of transgenic plants by increasing the ion flux, germination rates, chlorophyll content and antioxidant enzyme activities in roots and decreasing the electric conductivity and MDA content [143]. Saibi et al. [156] reported that the transgenic *A. thaliana* lines ectopically expressing the wheat *dehydrin* (*DHN*-5) gene revealed better SS tolerance by increasing the level of proline biosynthesis-related enzyme (P5CS), activating antioxidant enzymes and lowering H_2_O_2_ levels. Additionally, the introduction of the *PnF3H* gene from *Pohlia nutans* to *Arabidopsis* showed enhanced tolerance of transgenic plants to SS and oxidative stress. The expression of stress-related genes [e.g., *AtCAT1*, *AtAPX1*, *AtP5CS1* and *high-affinity K+ transporter* (*AtHKT1*)] were up-regulated, and activities of antioxidant enzymes were increased in the transgenic *Arabidopsis* plants [157] (Figure 2, left branch; Table 3). Abebe et al. [155] showed that ectopic expression of the *mannitol-1-phosphate dehydrogenase* (*mtlD*) gene from *E. coli* in *T. aestivum* exhibited enhanced tolerance to SS and water stress in terms of growth performance via biosynthesis of mannitol. *Myo-inositol-1-phosphate synthase* (*MIPS*) gene encoding a key rate-limiting enzyme in myo-inositol biosynthesis has been considered as one of the most important genes to improve tolerance to abiotic and biotic stresses [163]. Overexpression of *IbMIPS1* gene in sweet potato (*Ipomoea batatas*) significantly improved SS and drought tolerance, as well as stem nematode resistance, in transgenic plants [163]. The contents of MDA, H_2_O_2_ and Na^+^ significantly decreased, whereas the levels of inositol, phosphatidic acid, Ca^2+^, K^+^, abscisic acid, trehalose and proline significantly increased in the transgenic *I*. *batatas* plants under SS and drought [163].

Introduction of the *betaine aldehyde dehydrogenase* (*BADH*) gene from *Atriplex canescens* into potato enhanced SS tolerance of transgenic plants by increasing proline and chlorophyll contents, while lowering the contents of MDA and H_2_O_2_ and relative electric conductivity [160]. Ectopic expression of *Spinacia oleracea BADH* gene in Persian walnut (*Juglans regia*) improved the growth of transgenic plants under SS [161]. Wen et al. [162] showed that ectopic expression of the *spermidine synthase* (*MdSPDS1*) gene from apple (*Malus domestica*) in pear (*Pyrus communis*) enhanced tolerance to salt, osmotic and heavy metal stresses. Additionally, overexpression of *myo-inositol-1-phosphate synthase 1* (*MdMIPS1*) enhanced SS tolerance of transgenic apple plants by maintaining ion and osmotic balance, and improving the activities of antioxidant systems [164]. Japanese persimmon (*Diospyros kaki* Thunb. cv Jiro) was transformed with a gene from apple encoding *sorbitol-6-phosphate dehydrogenase* displayed enhanced SS by accumulation of sorbitol in transgenic plants [159].

S-adenosylmethionine decarboxylase (SAMDC) is a key enzyme in PA biosynthesis, and it plays an important role in plant responses to different ESs [165]. Ectopic expression of *SAMDC* gene of *Tritordeum* in rice increased the levels of Spd and Spm and enhanced SS tolerance of transgenic plants [151]. Ectopic expression of two bifunctional fusion genes (*OtsA* and *OtsB*) from *E. coli* in rice exhibited high trehalose accumulation and improved tolerance of transgenic plants to various ASs, including SS [153]. A similar type of work done by Li et al. [154], who overexpressed the *OsTPS1* gene in rice and reported tolerance of transgenic plants to SS and drought. Under these stress conditions, trehalose and proline contents were increased and the expression of some stress-related genes [e.g., *heat shock protein* (*HSP70*), *water stress inducible protein* (*WSI18*), *early light-inducible protein* (*ELIP*) and *responsive to ABA* (*RAB16C)*] were up-regulated in transgenic rice plants [154]. Walia et al. [166] reported that the expression of *CHI*, *F3′H* and *dihydroflavonol 4-reductase* (*DFR*) genes was up-regulated in the rice SS-sensitive genotype IR29 as compared with the SS-tolerant genotype FL478. Martinez et al. [62] noted that the expression of key genes *CHS* and *CHI* was up-regulated in *S. lycopersicon* under salinity, heat and combined stresses. Pi et al. [100] compared the salt tolerance levels of transgenic soybean roots overexpressing the *35S* promoter-driven coding sequence and RNAi constructs of *GmMYB173* and *GmCHS5*, as well as phospho-mimic (*GmMYB173_S59D_*) and phospho-ablative (*GmMYB173_S59A_*) versions of *GmMYB173* in relation to flavonoid accumulation. The authors reported that overexpression of *GmMYB173_S59D_* and *GmCHS5* conferred SS tolerance and accumulation of cyaniding-3-arabinoside chloride, a dihydroxy B-ring-substituted flavonoid. Studies investigating the transcription of key genes involved in the metabolism of phenolics in plants under SS have provided useful information. The above studies illustrate the potential of metabolic genes in engineering the crops for improved SS tolerance. The recent development in genetic engineering includes the discovery of metabolic genes related to SS tolerance [16,167,168]. Therefore, improving SS tolerance using genetic engineering approaches has attracted more studies.

## 5. Concluding Remarks and Future Perspectives

Over the last decade, different strategies have been used for enhancing ES tolerance, including SS based on metabolomic studies, use of priming agents and genetic manipulation. The applications of metabolomics in various studies have improved our knowledge of the metabolite constituents in many plant species, including the metabolic changes that occur in plants under ESs. The plant response/acclimation to SS through the changes of PMs and SMs enable plants to maintain their minimal growth under such limited conditions. Many studies (e.g., Table 1 and Table 2) have shown that PMs and SMs are involved in regulation of SS responses. The actions of metabolic compounds, such as trehalose, proline, GB, melatonin and PAs, have been earlier recognized as effective priming agents against SS. The utilization of priming agents as protectants to enhance SS tolerance in plants is extremely promising. Furthermore, genetic manipulation based on altering the expression levels of metabolic genes to maintain the function and structure of cellular components has also proven to be a promising approach for improving SS tolerance in plants. Both the approaches can modulate those metabolic and regulatory genes in crop plants and increase their SS tolerance. A new and novel approach for manipulation of the capacity of the plant immune system in combination with other approaches might hold the potential to achieve better protection of crop plants. The progress of molecular genetics has interestingly identified genes related to plant SS responses. Therefore, genetic engineering coupled with gene discovery will be endlessly continuing as a potential method for enhancing SS resistance by maintaining the structure and function of cellular components. The discovery of the CRISPR/Cas system has provided novel source of genetic diversity for breeding in a unique way. Priming agents, including some of the PMs or SMs, and genetic engineering will, therefore, continue to play an important role in plant response and adaptation to SS. The integrated application of multiple “omics” technologies, e.g., genomics, transcriptomics, proteomics and metabolomics, in a systematic manner is necessary to understand the molecular networks underlying plant responses to SS, as well as other types of ESs, which will allow us to develop crop varieties with better performance, and consequently yield improvement under different adverse environmental conditions to feed an ever-increasing global population.

## Figures and Tables

**Table 1:** Identification of important up-regulated and down-regulated metabolites in various plant species in response to salt stress.

Plant Species	Tissue	Methods of Analysis	Up-Regulated Metabolites during Salt Stress	Down-Regulated Metabolites during Salt Stress	References
*Aeluropus* *Lagopoides*	Shoots	CE-MS	**AA:** Alanine, asparagine, lysine, glutamine, arginine, glycine, proline, histidine, phenylalanine, serine, valine, tyrosine and cytosine **OM:** Adenine, adenosine and adenosine 5′-monophosphate	**OM:** Citrate, aconitate, 2-oxoglutarate, succinate, fumarate, ribulose-5-phosphate, ribose-5- phosphate and glucose-6-phosphate	[52]
*Hordeum. vulgare*	Leaves and roots	HPLC	**In roots:** **CH:** Sorbitol, mannitol and pinitol **In leaves:** **AA:** Proline **OM:** Glycine betaine	**In roots:** **OM:** Glycine betaine (undetectable) **In leaves:** **CH:** Sorbitol, mannitol and pinitol	[56]
*H. spontaneum,* *H. vulgare*	Leaves and roots	GC-MS	**In roots:** **CH:** Sucrose, trehalose, turanose, inositol, mannitol and xylitol AA: Proline **OM:** Citric acid and isocitric acid **In leaves:** **CH:** Isomaltose, raffinose and glucose **AA:** Asparagine, glycine, isoleucine, leucine, proline and serine **OM:** Pipecolic acid, glucose-6-phospate, fructose-6-phospate, 3-phosphoglyceric acid, pyruvate, uracil, diethylphosphate and putrescine	**In roots:** **AA:** Asparagine, β-alanine and valine **OM:** Fructose-6- phospate, glucose-6- phosphate, 3-phosphoglyceric acid, α-ketoglutaric acid, ascorbic acid, gluconic acid, orotic acid, pyroglutamic acid and threonic acid **In leaves:** **CH:** Sucrose, maltose, mannitol, glycerol and inositol **FA:** Palmitic acid **OM:** Citric acid, α-ketoglutaric acid, fumaric acid, malic acid, succinic acid, glucosan, menthol, 4-aminobutyrate, ascorbic acid, benzoic acid, pyroglutamic acid and threonic acid	[44]
*Zea mays*	Shoots and roots	^1^H-NMR	**In shoots:** **CH:** Sucrose **AA:** Alanine, glutamate and asparagine **OM:** Glycine betaine **In roots:** **CH:** Sucrose **AA:** Alanine **OM:** γ-amino-N-butyric acid, malic acid and succinate	**In shoots:** **CH:** Glucose **OM:** Citrate, malic acid, 2-oxoglutarate and *trans*-aconitic acid **In roots:** **CH:** Glucose **OM:** Acetoacetate	[57]
*Arabidopsis* *thaliana*	Roots and shoots	GC-MS	**CH:** Galactose, sucrose, trehalose, gentiobiose, melibiose, xylitol, galactinol, galactonate and threonate **AA:** Threonine, β-alanine, 5-oxoproline, glutamic acid, asparagine and glutamine **OM:** Glycerol-3-phosphate and *myo*-inositol-1-phosphate	**CH:** Fructose, erythritol and xylitol **AA:** Isoleucine and proline **OM:** Sinapinate, urea, succinate, citrate, aconitate, fumarate, malate, glycerate, propanoate and butyrate	[58]
*A. thaliana*	Whole Plant	GC-HP5890	**CH:** Sucrose, fructose, sorbose, raffinose and inositol **AA:** Glycine, proline serine, glutamic acid and threonine **OM:** Citric acid, galactinol, malic acid and phosphoric acid	**CH:** Glucose, glycerol, maltose and trehalose **AA:** Aspartic acid **OM:** Fumaric acid and succinic acid	[59]
*Lepidium latifolium*	Shoots and roots	HPLC and ^1^H-NMR	**In shoots:** **CH:** Sucrose, fructose, glucose, meso-inositol and chiro-inositol **AA:** Proline **In roots:** **CH:** Sucrose, fructose, glucose, meso-inositol and chiro-inositol **AA:** Proline and β-alanine **OM:** Choline-O-sulfate and β-alanine betaine	**In shoots:** **AA:** β-Alanine, glutamic acid and glutamine **OM:** Choline, choline-O-sulfate, β-alanine betaine, malate and citrate **In roots:** **AA:** Glutamic acid and glutamine **OM:** Choline, malate and citrate	[60]
*Thellungiella* *halophila*	Whole plant	GC-HP5890	**CH:** Fructose, sorbose, galactinol, glucose, glycerol, inositol, raffinose and trehalose **AA:** Aspartic acid glutamic acid, proline, glycine, serine and threonine	**CH:** Sucrose and maltose **FA:** palmitic acid and stearic acid **OM:** Citric acid, fumaric acid, malic acid and phosphoric acid	[59]
*Cicer arietinum*	Flower and pod tissues	GC-QqQ-MS and LC-MS	**CH:** Gentiobiose, fructose, sucrose and erythritol **AA:** Arginine, glutamic acid, glycine, histidine, homoserine, hydroxyproline, isoleucine, leucine, lysine, methionine, proline, threonine, tryptophan and valine **OM:** Pipecolate, isocitrate, *cis*-aconitate, citrate, fumarate, malate, citrate and 2-oxoglutarate	**CH:** Arabinose, erythritol and inositol **AA:** Cysteine **OM:** Putrescine and GABA	[45]
*Glycine max,* *G. soja*	Leaves	GC-MS and LC-FT/MS	**CH:** Lactitol and maltitol **FA:** Linolenic acid **OM:** Abscisic acid and caffeic acid	**CH:** Sucrose **AA:** Alanine **OM:** Glutathione	[51]
*Nicotiana tabacum*	Aerial Part	^1^H-NMR	**CH:** Glucose, fructose, sucrose and *myo*-inositol **AA:** Glutamine, proline, asparagine, valine, isoleucine, phenylalanine, tryptophan and tyrosine **OM:** Succinate, nicotine, formate and allantoin	**AA:** Aspartate and alanine **OM:** Malate, γ-amino-n-butyrate, choline, ethanolamine, hypoxanthine, dimethylamine, N-methylnicotinamide, uracil and uridine	[61]
*Solanum lycopersicon*	Leaves	UHPLC-QTOF-MS	**SM:** 1,3-dicaffeoylquinic acid, 1-feruoyl-5-caffeoylquinic acid, 3-caffeoyl-1-5 quinolactone and quercetin-3-hexodide	**SM:** Kaempferol, dihydrokaempferol, kaempferol 3-O-glucoside, naringenin, naringenin chalcone and quercetin-3-rutenoside	[62]
*Cuminum. cyminum*	Shoots	GC-MS and HPLC	**AA:** Isoleucine, glycine, proline, leucine and glutamate **FA:** Myristic acid, pentadeconoic acid, heptadecanoic acid, oleic acid, *cis*-11,14,17-eicosadienoic acid, heneicosanoic acid, palmitic acid, stearic acid and *cis*-11,14-eicosadienoic acid	**FA:** α-linolenic acid, *cis*-11-eicosadienoic acid, behenic acid, tricosanoic acid and linoleic acid	[9]
*Matricaria chamomilla*	Leaves and roots	HPLC	**In rosette leaves:** **AA:** Alanine, proline and tyrosine **OM:** Salicylic acid **SM:** protocatechuic, *p*-hydroxybenzoic ferulic acid, *o*-coumaric and *p*-coumaric **In roots:** **AA:** Alanine and proline **SM:** Chlorogenic, caffeic acid and *p*-coumaric	**In rosette leaves:** **AA:** Glutamic acid, serine, cysteine and lysine **SM:** Salicylic acid, chlorogenic acid and caffeic acid **In roots:** **AA:** Aspartic acid, glycine and phenylalanine **SM:** Benzoic acid, protocatechuic, protocatechuic, *p*-hydroxybenzoic aldehyde and *o*-coumaric	[63]
*Fagopyrum esculentum*	Sprouts	UFLC	**SM:** Isoorientin, orientin, rutin and vitexin		[64]
*Lotus* sp.	Shoots	GC/EI-TOF-MS	**CH:** Sucrose, *myo*-inositol and fructose **AA:** Proline, threonine, serine, glycine and phenylalanine **OM:** Glycerophosphoglycerol	**OM:** Citric acid, malic acid, succinic, fumaric, erythronic, glycolic and aconitic acid	[65]
*Nitraria tangutorum*	Cell suspensions	GC-MS	**AA:** Alanine, valine, serine, proline and asparagine **FA:** Hexadecanoic acid, octadecanoic acid and 9,12-octadecadienoic acid **OM:** Galactofuranose and succinate **SM:** Sitosterol	**OM:** Malic acid and acetamide	[66]
*Olea europaea*	Leaves and roots	HPLC	**SM:** Oleuropein	**SM:** Hydroxytyrosol	[67]
*Rosmarinus officinalis*	Leaves	HPLC	**SM:** Cineole and camphor	**SM:** Nopol, α-terpineol, borneol and camphene	[68]
*Salvia mirzayanii*	Leaves	HGC-MS	**OM:** α-terpinyl acetate, 1,8-cineole and linalyl acetate	**OM:** Bicyclogermacrene	[69]
*Salicornia brachiata*	Shoots	GC-MS, HPLC	**AA:** Proline, valine, isoleucine, leucine, cysteine, methionine and tyrosine **FA:** Tridecanoic acid, heptadecanoic acid, stearic acid, oleic acid, linoleic acid, α-linolenic acid, arachidic acid, heneicosanoic acid and lignoceric acid	**AA:** Glycine, arginine and serine **OM:** Myristoleic acid, pentadecanoic acid, palmitic acid and palmitoleic acid	[28]
*Atriplex. halimus*	Seedling	GC-FID and UPLC	**CH:** Saccharose and *myo*-inositol **AA:** Alanine, proline, arginine threonine, glycine, valine, leucine, phenylalanine and tryptophan	**CH:** Malate	[46]
*Suaeda corniculata*	Leaves	^1^H-NMR	**OM:** Betaine	**CH:** Sucrose, glucose and fructose **AA:** Valine, glycine, alanine, leucine, isoleucine, glutamine, glutamate, aspartate and threonine **OM:** Malate, succinate, 2-oxoglutarate, fumarate, dimethylamine and choline	[53]
*Suaeda maritima*	Shoots and roots	GC-FID, UPLC and ^1^H-NMR	**CH:** Sucrose **AA:** Proline, valine, glutamine and glycine **OM:** Betaine, citrate and glycerate	**CH:** Glucose, fructose and *myo*-nositol **AA:** Serine, glutamate isoleucine and threonine **OM:** GABA	[70]

AA, amino acid; CE-MS, capillary electrophoresis-mass spectrometry; CH: carbohydrate; EI, electrospray ionization; FA, fatty acid; GABA, γ-aminobutyric acid; GC-HP5890, gas chromatography-Hewlett packard 5890; GC-MS, gas chromatography-mass spectrometry; GC-QqQ-MS, gas chromatography-triple quadrupole-mass spectrometry; GC-FID, gas chromatography-flame ionization detector; HGC-MS, headspace gas chromatography-mass spectrophotometry; HPLC, high-performance liquid chromatography; LC-FT/MS, liquid chromatography-fourier transform-mass spectrometry; LC-MS, liquid chromatography-mass spectrometry; ^1^H-NMR, nuclear magnetic resonance; OM, other metabolites; SM, secondary metabolites; TOF, time-of-flight; UPLC, ultra-performance liquid chromatography; UHPLC-QTOF-MS, ultra-high performance liquid chromatography-quadrupole time-of-flight mass spectrometry; UFLC, ultra-fast liquid chromatography.

**Table 2:** Effects of exogenous metabolites on the improvement of salt tolerance of treated plants.

Metabolites	Plant Species	Stress Tolerance	References
Proline and trehalose	*Oryza sativa*	Salt stress	[119]
Salicylic acid	*O. sativa*	Salt stress	[120]
Spermidine or spermine	*O. sativa*	Salt stress	[121]
Glycine betaine	*O. sativa*	Salt stress	[122]
Sorghum extracts and benzyl aminopurine	*Triticum aestivum*	Salt stress	[123]
Methyl jasmonate	*T. aestivum*	Salt stress	[124]
Spermidine	*Zoysia japonica*	Salt stress	[125]
5-aminolevulinic acid	*Brassica napus*	Salt stress	[126]
Allantoin	*Arabidopsis thaliana*	Salt stress	[115]
γ-aminobutyrate	*Cucumis melo*	Saline- alkaline stress	[127]
Glutathione	*Solanum lycopersicum*	Salt stress	[128]
Omeprazole	*S. lycopersicum*	Salt stress	[129]
Penconazole	*Carthamus tinctorius*	Salt stress	[130]
Putrescine	*C. sativus*	Salt stress	[131]
Melatonin	*Citrullus lanatus*; *T. aestivum*; *S. lycopersicum*; *C. sativus; Helianthus annuus*; *O. sativa*; *A. thaliana*; *Zea mays*; *B. napus*	Salt stress	[116,117,118,132,133,134,135,136,137]
Proline	*Z. mays*; *Sorghum bicolor*	Salt stress	[138,139]

**Table 3:** Improvements of plant tolerance to salt stress by genetic engineering of metabolic genes.

Gene	Locus ID	Source	Transgenic Plants	Stress Tolerance	References
*∆^1^-pyrroline-5-carboxylate synthetase* (*P5CS*)	VIRPYRR	*Vigna aconitifolia*	*Oryza sativa*	Salt stress	[144]
*∆^1^-pyrroline-5-carboxylate synthetase* (*P5CSF129A*)	P5CS_VIGAC	*V. aconitifolia*	*O. sativa*	Salt stress	[146]
*S-adenosylmethionine decarboxylase* (*SAMDC*)	CAA58762	*Tritordeum*	*O. sativa*	Salt stress	[151]
*Trehalose-6-phosphate synthase (TPS)* and *trehalose-6-phosphate phosphatase (TPP)*	EU070413 and NC_002695	*Escherichia coli*	*O. sativa*	Salt stress	[152]
*Trehalose-6-phosphate synthase (TPS; otsA)* and *Trehalose-6-phosphate phosphatase (TPP; otsB)*	NC_000913	*E. coli*	*O. sativa*	Salinity, cold (10 °C) and dehydration	[153]
*Trehalose-6-phosphate synthase1*	HM050424	*O. sativa*	*O. sativa*	Salinity and polyethylene glycol	[154]
*Choline dehydrogenase (betA)*	NC_000913	*E. coli*	*Triticum aestivum*	Salt stress	[149]
*Mannitol-1-phosphate dehydrogenase* (*mtlD*)	EFF7369098	*E. coli*	*T. aestivum*	Salinity and polyethylene glycol	[155]
*Cysteine protease* (*SmCP*)	KC715825	*Salix matsudana*	*Arabidopsis*	Salt stress	[143]
*Dehydrin (DHN-5)*	CAY85463	*T. aestivum*	*Arabidopsis*	Salt stress	[156]
*Flavanone 3-hydroxylase* (*PnF3H*)	MK036761	*Pohlia nutans*	*Arabidopsis*	Salinity and oxidative stress	[157]
*Choline oxidase* (*codA*)	AY304485	*Arthrobacter. globiformis*	*Diospyros kaki*	Salt stress	[158]
*Sorbitol-6-phosphate dehydrogenase* (*S6PDH*)	NM_001294028	*Malus domestica*	*D. kaki*	Salt stress	[159]
*Arginine decarboxylase* (*ADC*)	BQ739966	*Avena sativa*	*Lotus tenuis*	Salt stress	[147]
*Choline monooxygenase* (*AhCMO*)	AF270651	*Atriplex hortensis*	*Gossypium hirsutum*	Salt stress	[150]
*Betaine aldehyde dehydrogenase (BADH)*	JF776157	*A. canescens*	*Solanum tuberosum*	Salt stress	[160]
*Betaine aldehyde dehydrogenase (BADH)*	FJ595952	*Spinacia oleracea*	*Juglans regia*	salinity and drought	[161]
*Spermidine synthase (MdSPDS1)*	LOC103451952	*M. domestica*	*Pyrus communis*	Salt, osmotic and copper stresses	[162]

## Abbreviations

ASs	Abiotic stresses
AAs	Amino acids
ALA	5-aminolevulinic acid
APX	Ascorbate peroxidase
CAT	Catalase
DMSP	Dimethylsulfoniopropionate
DMSO	Dimethylsulfoxide
ESs	Environmental stresses
GB	Glycine betaine
GABA	γ-amino butyric acid
MDA	Malondialdehyde
PMs	Primary metabolites
PA	Polyamines
Put	Putrescine
ROS	Reactive oxygen species
SMs	Secondary metabolites
SS	Salt stress
SOD	Superoxide dismutase
Spd	Spermidine
Spm	Spermine
